# Neuroendocrine tumor of the small intestine: case report

**DOI:** 10.1590/0102-672020190001e1492

**Published:** 2020-05-18

**Authors:** Douglas Jun KAMEI, Rafael Shinmi SHIGUIHARA, Fernando Romani de ARAÚJO

**Affiliations:** 1 Department of General and Digestive Surgery, Santa Casa de Curitiba Hospital, Curitiba, PR, Brazil

**Keywords:** Neuroendocrine tumor, Carcinoid tumor, Carcinoid syndrome, Small intestine, Hepatic metastasis, Tumor neuroendócrino, Tumor carcinoide, Síndrome carcinoide, Intestino Delgado, Metástase hepática

## INTRODUCTION

The neuroendocrine tumor, also known as carcinoid tumor, is a neoplasm of the diffuse neuroendocrine system^4^. The occurrence of this type of tumor in the small intestine is rare and has a genetic influence in its etiology. It has been estimated that the deletion of the tumor suppressor gene PLCβ3 causes the uncontrolled growth of the neuroendocrine cells^2^ ^,^ ^4^.The incidence ranges from 1 to 2 per 100,000 and affects men and women equally. Most of these tumors are well-differentiated and have an indolent course. Consequently, the onset of symptoms is late and, in most cases, the diagnosis is made at advanced stages of the disease. The chosen therapeutics is tumor resection. For the initial stages, the method aims at healing; while in advanced phases, the cytoreductive operation, associated to the multidisciplinary treatment, provides an increase of the survival time^9^. 

The objective of this study was to present a case of a metastatic neuroendocrine tumor of small intestine with a characteristic course of carcinoid syndrome.

## CASE REPORT

**FIGURE 1:**
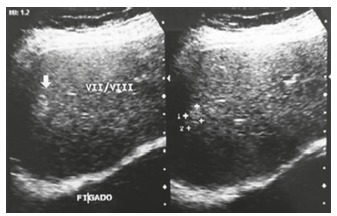
Ultrasonography with nodular image suggestive of hemangioma in segment VII/VIII

A 57-year-old woman, referred to Hospital Santa Casa in Curitiba, PR, Brazil due to the presence of hepatic nodules that suggested of hemangiomas on abdominal ultrasonography (Figure 1) and diarrhea episodes with sporadic facial flushes.

**FIGURE 2:**
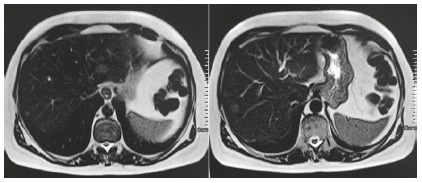
To the left there is resonance imaging showing hyperintense hepatic nodule in segment II, and to the right a hyperintense nodule in segment VIII.

During ambulatory investigation, the presence of 15 mm solid liver nodules in segments II and VIII was confirmed by computed tomography of the abdomen, with evidence of contrast washout in the late arterial phase. Magnetic resonance imaging (Figure 2) showed, in addition to nodules in segments II and VIII, other 5 mm nodules in segments V and VI. Due to the suspected malignancy of the hepatic tumor; the patient underwent biopsy of the liver, omentum and peritoneum.

The omentum and peritoneum presented no neoplasia. In hepatic tissue, the nature of the lesion was confirmed by immunohistochemistry, that showed positivity for synaptophysin (clone SY38), chromogranin A (clone DAK-A3), CDX-2, AE1/AE3 diffusely and KI-67 positive on 2% of cells. The immunohistochemical panel was accordant with an infiltrative and well differentiated neuroendocrine tumor in the liver, with its origin in the digestive tract. After the biopsy results, was requested a 24 h urine 5-hydroxy-indolacetic acid test.

**FIGURE 3:**
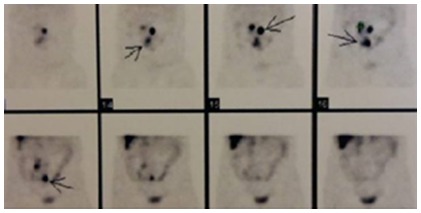
Octreoscan with high uptake areas of somatostatin analogue in the abdomen with mesenteric lymph node metastasis

Through computed tomography enterography and OctreoScan (Figure 3) was, respectively, confirmed the presence of polypoid lesions in the ileal region and mesenteric lymph node metastasis. The patient underwent an intraoperative enteroscopy and a tumor resection in the small intestine, with the removal of 80 cm of ileum and the realization of an enteroenteroanastomosis. Furthermore, were removed nine lymphnodes. She has evolved well in the postoperative period and was discharged with a prescription of Sandostatin LAR (Octreotide 20 mg at 4-week intervals).

The product of the enterectomy presented seven infiltrative nodular formations at macroscopy and, in the anatomopathological exam, the result founded indicated a well differentiated epithelioid neoplasia with the characteristics of neuroendocrine infiltration in multiple locations. Seven out of the nine lymph nodes were compromised. While in the chest computed tomography no particularities were observed.

After one year postoperatively, was observed a significant improvement. After six applications of Sandostatin LAR, the symptoms were reduced, from two episodes of diarrhea per day and sporadic facial flushes to only one diarrhea episode and no facial flushes every two weeks. The results of the 24 h urine 5-hydroxy-indolacetic acid test presented significant decrease as well. Before surgery and octreotide, the values were 71.3 mg, which reduced to 6.9 mg on the following postoperative and octreotide analysis. The patient remains under medical monitoring to assess the possibility of the hepatic metastasis resection. 

## DISCUSSION

The neuroendocrine tumors affect the diffuse endocrine system, which is formed by small groups of cells distributed throughout the body. The main concentration of these cells is at the gastroenteropancreatic tissues, specially the intestinal mucosa and submucosa. They can be found also in respiratory system, thymus, urogenital system and skin^6^. Despite the major intestine cell clustering, the small intestine neuroendocrine tumor is considered a rare entity^2^ ^,^ ^6^.

These tumors present the characteristics of synthesize and secrete peptides e amines. When such substances are released and activated, they generate a clinical syndrome. However, when these tumors secrete non-active substances or don’t secrete them at all, they course with a mass-effect syndrome^1^ ^,^ ^6^. 

A carcinoid syndrome, as it is known the clinical syndrome, is composed by a series of symptoms such as diarrhea, facial flushes, bronchospasm, cyanosis and inconstancy of the blood pressure as a result of the serotonin production. The syndrome affects around 5-7% of patients^1^ ^,^ ^2^ ^,^ ^10^. In this case report, the patient represents the minority group that is affected by the clinical syndrome. 

The chronic course is fairly typical of these tumors. Some patients might present less specific symptoms, like abdominal pain, small enterorrhages and intestinal obstruction. When such symptoms and the carcinoid syndrome are present, 12% of the individuals already show distant metastasis, mainly in the liver^6^ ^,^ ^10^. 

Regarding the hepatic metastasis, there is no specific protocol for therapeutics. Some studies argue that, in case of unresectable metastasis, the use of somatostatin analogs along with cytoreduction surgery promotes an increase in patient survival and an improvement in quality of life^3^ ^,^ ^5^. The use of somatostatin analogues as a method of prevention of the carcinoid crises remains very controversial. The study made by Guo et al^8^ argue that these medications are inefficient. However, Gregersen et al^7^ concluded that, in addition to an improvement of the diarrheal episodes, the somatostatin analogs also reduce the serotonin, chromogranin A and urinary 5-hydroxyindoleacetic acid levels. For the correction of the primary site, radical resection of the neuroendocrine tumor is the therapy of choice^2^. In the present case, the approach chosen was the radical resection and the use of Sandostatin Lar, with a significant improvement in the clinical status and reduction of the urinary levels of 5-hydroxyindoleacetic acid. 
